# Preconception use of cART by HIV-positive pregnant women increases the risk of infants being born small for gestational age

**DOI:** 10.1371/journal.pone.0191389

**Published:** 2018-01-19

**Authors:** Ingrid J. M. Snijdewind, Colette Smit, Mieke H. Godfried, Rachel Bakker, Jeannine F. J. B. Nellen, Vincent W. V. Jaddoe, Elisabeth van Leeuwen, Peter Reiss, Eric A. P. Steegers, Marchina E. van der Ende

**Affiliations:** 1 Department of Internal Medicine, Section Infectious Diseases, Erasmus Medical Center, Rotterdam, The Netherlands; 2 Stichting HIV Monitoring (SHM), Amsterdam, The Netherlands; 3 Department of Internal Medicine and Infectious Disease, Academic Medical Center, Amsterdam, The Netherlands; 4 Department of Epidemiology, Erasmus Medical Center, Rotterdam, the Netherlands; 5 Department of Paediatrics, Erasmus Medical Center, Rotterdam, the Netherlands; 6 Department of Obstetrics and Gynaecology, Academic Medical Center, Amsterdam, The Netherlands; 7 Department of Global Health, Academic Medical Center and Amsterdam Institute for Global Health and Development, Amsterdam, The Netherlands; 8 Department of Obstetrics and Gynaecology, Erasmus Medical Center, Rotterdam, The Netherlands; Stellenbosch University, SOUTH AFRICA

## Abstract

**Background:**

The benefits of combination anti-retroviral therapy (cART) in HIV-positive pregnant women (improved maternal health and prevention of mother to child transmission [pMTCT]) currently outweigh the adverse effects due to cART. As the variety of cART increases, however, the question arises as to which type of cART is safest for pregnant women and women of childbearing age. We studied the effect of timing and exposure to different classes of cART on adverse birth outcomes in a large HIV cohort in the Netherlands.

**Materials and methods:**

We included singleton HEU infants registered in the ATHENA cohort from 1997 to 2015. Multivariate logistic regression analysis for single and multiple pregnancies was used to evaluate predictors of small for gestational age (SGA, birth weight <10^th^ percentile for gestational age), low birth weight and preterm delivery.

**Results:**

A total of 1392 children born to 1022 mothers were included. Of these, 331 (23.8%) children were SGA. Women starting cART before conception had an increased risk of having a SGA infant compared to women starting cART after conception (OR 1.35, 95% CI 1.03−1.77, p = 0.03). The risk for SGA was highest in women who started a protease inhibitor-(PI) based regimen prior to pregnancy, compared with women who initiated PI-based cART during pregnancy. While the association of preterm delivery and preconception cART was significant in univariate analysis, on multivariate analysis only a non-significant trend was observed (OR 1.39, 95% CI 0.94−1.92, p = 0.06) in women who had started cART before compared to after conception. In multivariate analysis, the risk of low birth weight (OR 1.34, 95% CI 0.94−1.92, p = 0.11) was not significantly increased in women who had started cART prior to conception compared to after conception.

**Conclusion:**

In our cohort of pregnant HIV-positive women, the use of cART prior to conception, most notably a PI-based regimen, was associated with intrauterine growth restriction resulting in SGA. Data showed a non-significant trend in the risk of PTD associated with preconception use of cART compared to its use after conception. More studies are needed with regard to the mechanisms taking place in the placenta during fetal growth in pregnant HIV-positive women using cART. It will only be with this knowledge that we can begin to understand the potential impact of HIV and cART on the fetus, in order to be able to determine the optimal individualised drug regimen for HIV-infected women of childbearing age.

## Introduction

The benefits of combination anti-retroviral therapy (cART) in HIV-positive pregnant women (improved maternal health and prevention of mother to child transmission [pMTCT]) currently outweigh the adverse effects due to cART. As the variety of cART increases, however, the question arises as to which type of cART is safest for pregnant women and women of childbearing age. Most cART studies in HIV-positive women and their HIV-exposed uninfected (HEU) infants, however, have focused on adverse birth outcomes, such as preterm delivery (PTD) and low birth weight (LBW). As yet, any association between maternal exposure to cART, (including exposure to protease inhibitors [PI]), and the timing of initiation of cART) and adverse outcomes remains unclear [1–6]. Furthermore, studies exploring the relationship between cART exposure during pregnancy and the occurrence of small for gestational age (SGA) births are scarce [7–11], while studies describing *in utero* exposure to cART and fetal growth and child development have been inconclusive. Some studies have found that the use of cART prior to conception increases the risk of PTD [9, 12, 13] and LBW [5, 14, 15] however, as the definition of LBW is an infant weighing <2.5 kg, it is not possible to distinguish between preterm and SGA infants.

Evaluating fetal growth is complex, involving the consideration of race, cigarette smoking, nutritional status, disease stage of HIV and exposure to cART. In the Netherlands, all pregnant women have equal access to (ante-natal) care and, since 1997, all HIV-positive pregnant women have been offered cART, usually from the second trimester onwards. Women who were already receiving cART prior to conception, are encouraged to continue with anti-retroviral treatment, unless there are concerns regarding teratogenicity and virological failure, or if they are unable to tolerate the cART regimen (which may, therefore, need to be modified).

Our objective for this study was to investigate whether or not there may be an association between different types of cART (and the timing of its initiation) and the occurrence of SGA in HEU infants.

## Materials and methods

### Study type

This was a retrospective, observational study; pregnancy-related data were obtained from an ongoing observational HIV cohort study.

### Population

Data were obtained from the HIV Monitoring Foundation ATHENA cohort database, which contains records from 1997 onwards, from all HIV-positive women receiving care at any of the 26 HIV treatment centers in the Netherlands. Written informed consent was obtained from all of the individuals concerned [16]. Only HIV-positive women (≥18 years of age) who gave birth to HEU infants after a minimum of 24 weeks’ pregnancy were eligible for the study. Singleton live-births (January 1997 to February 2015) were evaluated. This included HIV-positive women who had started cART comprising at least three antiviral drugs prior to conception, as well as HIV-positive women who began cART during pregnancy.

For comparison, we used data from the Generation R Study, a multi-ethnic, urban population-based prospective cohort study, conducted in Rotterdam, involving HIV-negative women and their children. Infants were followed from the first trimester of pregnancy up to young adulthood [17]. The Generation R women were very different to our population in relation to ethnicity (our population were mostly women from sub-Saharan Africa, SSA), substance abuse, obstetric follow-up and socioeconomic background. In order to provide a better match to our population, we only selected SSA women from the Generation R study, to illustrate growth potential in the Netherlands in children with a similar migration history.

### Variables

Baseline data collected from the women’s medical histories included maternal age at delivery, region of origin (West European, SSA, or other), and cigarette smoking, alcohol and illicit drug use during pregnancy. Pregnancy- and birth-related data comprised gestational age, assessed routinely by ultrasound during the first trimester or counted from the first day of the woman’s last menstrual period, fetal growth at 32 weeks’ gestation, parity and mode of delivery. Infant-related data comprised gender and birth weight. Medication-related data consisted of date of cART-initiation, duration of cART, and type of regimen. The women were receiving cART comprising three different drugs from two pharmacological classes. The clinical laboratory measurements comprised CD4^*+*^ cell count during pregnancy and closest to delivery (up to 6 months after delivery), and HIV-RNA during pregnancy, closest to delivery (up to 3 months after delivery).

Self-reporting variables included cigarette smoking, alcohol and illicit drug use. Mode of delivery was classified as spontaneous vaginal delivery, assisted vaginal delivery and elective or emergency Caesarean section. SGA was defined as infant birth weight below the 10th percentile, adjusted for gestational age, gender and parity according to Dutch non-Hindustani reference curves [18]. PTD was defined as birth at <37 weeks’ gestation and very preterm as <32 weeks’ gestation. In keeping with the literature, a birth weight above 2.5 kg was considered normal, LBW was <2.5 kg and below 1.5 kg was recorded as very low birth weight (VLBW). Elective Caesarean section in HIV-positive women, in The Netherlands, is usually scheduled at 38 weeks in cases of detectable viremia (virological failure) or late initiation of cART and after 39 weeks for other obstetric indications. An emergency Caesarean delivery was defined as a Caesarean delivery that was performed after the onset of labour. CD4^*+*^ cell count was split into three categories: <200 cells/μl, 200–500 cells/μl and >500 cells/μl. Plasma HIV-RNA levels were defined as undetectable at <500 copies/ml (to allow for inter-hospital variation and older sampling sensitivities).

### Study outcomes

The primary outcome was the proportion of HEU infants who were SGA. Secondary outcomes were: median birth weight (LBW or VLBW), gestational age in weeks, PTD or very PTD).

#### Statistical analyses

Mann-Whitney and Chi square tests were used to compare baseline characteristics. In order to assess the risk of SGA, a logistic regression model including generalized estimating equations (GEE) was used that accounted for multiple pregnancies in one woman. Independent variables were calculated separately using a univariate binary logistic regression analysis with GEE. Missing values in the equation were analysed as a group and taken into the univariate and multivariate models. All individual variables with a p-value of ≤0.10 in the univariate analyses were included in the multivariate logistic regression analysis. A probability value of <0.05 was considered statistically significant in the multivariate models. Statistical analyses were performed using the Statistical Package for Social Sciences version 21 software package for Windows (SPSS Inc, IBM, Chicago, IL, USA) and SAS^®^ 9.3 Software.

## Results

### Patients

A total of 2184 pregnancies among the HIV-positive women from the ATHENA cohort were registered between 1997 and 2015. Of these, 619 ended in miscarriage (n = 344; 15.8%), or elective termination (n = 258; 11.8%) and data were missing for 17 pregnancies. We excluded 23 twin pregnancies. Data on gestational age or birth weight were missing in 48 births, five births occurred before 24 weeks’ gestation and 82 women were either not using any anti-retroviral drugs, or were using an unknown regimen, or a regimen of fewer than three anti-retroviral drugs. A total of 15 women were <18 years of age when they gave birth.

In total, 1392 singleton births were included in the analysis. cART was used prior to and at the time of conception by 550 women. A total of 842 women were not using any anti-retroviral therapy at the time of conception, of whom 625 (74.2%) were cART-naïve and 217 women (25.8%) had previously received cART. Women who started cART prior to conception had been receiving cART for a median of 3.48 years (IQR 2.08–5.35).

### Baseline maternal characteristics

Baseline demographic, laboratory and treatment characteristics are shown in Table 1. The data were split into two groups: those women who had started cART prior to conception and those who had started after conception. In women who began cART after conception, we included both cART-naive and cART-experienced women who were drug-free at the time of this conception and during the first trimester.

**Table 1:** Baseline characteristics of HIV-negative and HIV-positive women (by group–those starting cART prior to conception and those starting cART after conception).

	HIV-negative Generation R	HIV-positive women (SHM)			P- Value*
	n = 9403 (%)	Total n = 1392 (%)	Preconception cART n = 550 (%)	Postconception cART n = 842 (%)
**Maternal age at childbirth (yrs)**					
Median	30.45	29.89	32.7	28.8	<0.001
IQR	26.09–33.70	25.75–34.36	28.4–36.3	25.1–33.1	
Missing		10 (0.7)	8 (1.5)	2 (0.2)	
**Region of origin**					
SSA	900 (9.6)	853 (61.3)	349 (63.5)	504 (59.9)	
W. Europe	6207 (66.0)	288 (20.7)	110 (20.0)	178 (21.1)	0.42
Other	2296 (24.4)	251 (18.0)	91 (16.5)	160 (19.0)	0.19
**Cigarette smoking status**					
Yes	1053 (11.2)	114 (8.2)	34 (6.2)	80 (9.5)	
No	1935 (20.6)	643 (46.2)	209 (38.0)	434 (51.5)	0.57
Unknown	6415 (68.2)	635 (45.6)	307 (55.8)	328 (39.0)	<0.001
**Alcohol use**					
Yes	1190 (12.7)	75 (5.4)	29 (5.3)	46 (5.5)	
No	6176 (65.7)	664 (47.7)	208 (37.8)	456 (54.2)	0.20
Unknown	2038 (21.7)	653 (46.9)	313 (56.9)	340 (40.4)	<0.001
**Illicit drug use**					
Yes	41 (0.4)	22 (1.6)	6 (1.1)	16 (1.9)	
No	8028 (85.4)	706 (50.7)	224 (40.7)	482 (57.2)	0.66
Unknown	1334 (14.2)	664 (47.7)	320 (58.2)	344 (40.9)	<0.001
**Parity**					
Primipara	2594 (27.6)	477 (34.3)	164 (29.8)	313 (37.2)	
Multipara	6573 (69.9)	915 (65.7)	386 (70.2)	529 (62.8)	0.01
Unknown	236 (2.5)				
**Mode of delivery**					
Spontaneous labour	7259 (77.2)	619 (44.5)	198 (36.0)	421 (50.0)	
Primary (elective) C-section	419 (4.5)	189 (13.6)	60 (10.9)	129 (15.3)	0.95
Secondary (emergency) C-section	643 (6.8)	198 (14.2)	71 (12.9)	127 (15.1)	0.31
Unknown	1082 (11.5)	386 (27.7)	221 (40.2)	165 (19.6)	<0.001
**BMI (kg/m**^**2**^**)**					
Median	23.87	24.9	23.6	25.2	0.07
IQR	21.71–27.06	21.9–28.4	21.4–26.9	22.5–29.1	
Unknown	1539 (9.89)	708 (50.9)	339 (61.6)	369 (43.8)	
**CD4**^***+***^ **(cells/μl)**					
Median		520	540	510	0.26
IQR		374–700	381.6–720.8	364–690	
Missing		24 (1.7)	14 (2.5)	10 (1.2)	
**Nadir CD4**^**+**^ **(cells/μl)**					
>500		258 (18.5)	26 (4.7)	232 (27.6)	
200–500		643 (46.2)	218 (39.6)	425 (50.5)	<0.001
<200		491 (35.3)	306 (55.6)	185 (22.0)	<0.001
**HIV RNA concentration (copies/ml)**					
≤500		1101 (79.1)	484 (88.0)	617 (73.3)	
>500		272 (19.5)	59 (10.7)	213 (25.3)	<0.001
Missing data		19 (1.4)	7 (1.3)	12 (1.4)	<0.001
**cART regimen**					
PI-based		928 (66.7)	269 (48.9)	659 (78.3)	
NNRTI-based		438 (31.5)	263 (47.8)	175 (20.8)	<0.001
Both or NRTI		12 (0.9)	4 (0.7)	8 (1.0)	<0.001

* P-values were calculated using Mann-Whitney and Chi square tests

Table 1 abbreviations

SHM: Stichting HIV Monitoring; cART: combination antiretroviral therapy; SSA: Sub-Saharan Africa; W. Europe: Western Europe; IQR: interquartile range; C-section: Caesarean section; BMI: body mass index; PI: Protease inhibitors; NNRTI: Non-nucleoside reverse-transcriptase inhibitors; Both: both PI- and NNRTI-based regimen; NRTI: nucleoside reverse transcriptase inhibitors.

HIV-negative (Generation R) Solely to describe the non-HIV population in the Netherlands we selected 9778 children from HIV-negative control women who gave birth and were included in the Generation R study from 2000 to 2006. Exclusion criteria were a termination of pregnancy (n = 29), intra uterine fetal death (n = 75; 0.7%), HIV-positivity (n = 29), twin pregnancies (n = 262), missing data on birth weight (n = 77), an unknown gestational age (n = 2) or postnatal inclusion (n = 765). A total of 8539 singleton live births after at least 24 weeks gestation were included in the tables.

HIV-positive women who started cART prior to conception were older (32.7 vs 28.8 years of age), more often multiparous (70.2% vs 62.8%), on a non-nucleoside reverse transcriptase inhibitor- (NNRTI) based regimen (47.8% vs 20.8%) and with a nadir CD4^+^ cell count of <200 cells/μl (55.6% vs 22.0%) compared to women who started cART after conception. Median baseline CD4^+^ cell count at the start of pregnancy in these HIV-positive women was similar between the two groups.

### Pregnancy outcome

#### SGA

The SGA rate among the population of HIV-negative women from Generation R was 1.8%; women from SSA had an SGA rate of 1.4% (S1 Table). In the current study, the overall rate of SGA in HEU infants was 23.8% (331/1392) (Table 2). Among the infants of women who started cART prior to conception, the SGA was 27.3% vs 21.5% in women who started cART after conception (p = 0.01) (see Table 2). Univariate SGA odds ratio was 1.40 (95% CI 1.11−1.80 p = 0.005) using a GEE. After correcting for cART regimen, region of origin and parity (variables with p≤0.1 in the univariate analysis), the risk for SGA was significantly higher among women who started cART before conception compared to those who only began cART after conception (1.35, 95% CI 1.03–1.77, p = 0.028) (Table 3). Stratifying for a PI- or NNRTI-based cART regimen, the multivariate analysis also indicated a significantly increased risk of SGA among mothers who used a PI-based regimen prior to conception compared with those who had used this regimen only after conception (OR 1.49, 95% CI 1.08−2.10, p = 0.016) (Table 4). We could not demonstrate this increased risk of SGA among the infants of women who started a NNRTI-based regimen prior to vs after conception (OR 0.97, 95% CI 0.62−1.52, p = 0.91) (Table 4).

**Table 2:** Outcome of HIV-exposed uninfected (HEU) singleton infants born to HIV-positive mothers by timing of initiation of cART—preconception vs postconception.

	HIV-positive women (SHM)			P- Value*
	Total n = 1392	Preconception cART n = 550	Postconception cART n = 842	
**SGA <10th (n, %)**				
No	1061 (76.2)	400 (72.7)	661 (78.5)	
Yes	331 (23.8)	150 (27.3)	181 (21.5)	0.01
**SGA <5th (n, %)**				
No	1199 (86.1)	457 (83.1)	742 (88.1)	
Yes	193 (13.9)	93 (16.9)	100 (11.9)	0.01
**Median birth weight (kg)**	3.090	3.070	3.103	0.22
IQR	2.702−3.405	2.637−3.384	2.754−3.441	
**Birth weight (n, %)**				
≥2.5 kg	1174 (84.3)	448 (81.5)	726 (86.2)	
1.5−2.5 kg	173 (12.4)	78 (14.2)	95 (11.3)	0.08
<1.5 kg	45 (3.2)	24 (4.4)	21 (2.5)	0.04
**Median duration of pregnancy (weeks)**	39.14	39.00	39.14	
IQR	38.00–40.14	37.71–40.14	38.00–40.29	0.23
**Duration of pregnancy (weeks)**				
>37	1188 (85.3)	454 (82.5)	734 (87.2)	
<37	165 (11.9)	74 (13.5)	91 (10.8)	0.09
<32	39 (2.8)	22 (4.0)	17 (2.0)	0.02
**Perinatal death (n, %)**				
No/unknown	1374 (98.7)	546 (99.3)	828 (98.3)	
Yes	18 (1.3)	4 (0.7)	14 (1.7)	0.30
**Gender**				
Male	717 (51.5)	283 (51.5)	434 (51.5)	
Female	674 (48.4)	267 (48.5)	407 (48.3)	0.96
Unknown	1 (0.1)		1 (0.1)	1.00

* P-values were calculated using chi square

Table 2 abbreviations

cART: combination antiretroviral therapy; SGA <10th: Small for gestational age <10th percentile; SGA <5th: Small for gestational age <5th percentile; IQR: interquartile range

**Table 3:** Risk of babies being born SGA <10th percentile, by univariate and multivariate analysis, using a generalized estimation equation.

	Total	SGA	Univariate analysis OR (95% CI)	P- Value*	Multivariate analysis OR (95% CI)	P- Value*
	1392	33 (%)				
**Initiation of cART**						
Postconception	842	181 (21.5)	1		1	
Preconception	550	150 (27.3)	1.40 (1.11–1.80)	0.01	1.35 (1.03–1.77)	0.03
**Type of cART regimen**						
PI-based	928	215 (23.2)	1		1	
NNRTI-based	438	105 (24.0)	1.04 (0.80–1.16)	0.76	0.95 (0.71–1.27)	0.73
Both/NRTI only	25	11 (44.0)	2.51 (1.16–5.53)	0.02	2.11 (0.98–4.57)	0.06
**Age at delivery**						
			1.01 (0.99–1.04)	0.21		
**BMI**						
			0.99 (0.95–1.03)	0.59		
**Maternal CD4**^*+*^ **concentration at delivery (cells/μl)**						
≥500	738	181 (24.5)	1			
200−500	570	128 (22.5)	0.89 (0.69–1.16)	0.39		
<200	60	15 (25.0)	0.97 (0.53–1.77)	0.91		
**Nadir maternal CD4**^**+**^ **concentration (cells/μl)**						
≥500	258	56 (21.7)	1			
200–500	643	156 (24.3)	1.17 (0.81–1.72)	0.39		
<200 cells	491	119 (24.2)	1.19 (0.80–1.75)	0.38		
**Region of origin**						
SSA	853	206 (24.2)	1		1	
W. Europe	288	55 (19.1)	0.73 (0.52–1.04)	0.08	0.76 (0.54–0.92)	0.13
Other	251	70 (27.9)	1.19 (0.85–1.85)	0.31	1.17 (0.84–1.63)	
**Smoking status**						
No	643	156 (24.3)	1			
Yes	114	27 (23.7)	0.94 (0.75–1.55)	0.68		
Unknown	635	148 (23.3)	0.98 (0.76–1.27)			
**Alcohol use**						
No	664	165 (24.8)	1			
Yes	75	18 (24.0)	0.95 (0.54–1.67)	0.40		
Unknown	653	148 (22.7)	0.92 (0.72–1.19)	0.46		
**Illicit drug use**						
No	718	188 (26.2)	1			
Yes	21	7 (33.3)	1.49 (0.56–3.74)	0.40		
Unknown	299	68 (22.7)	0.89 (0.64–1.23)	0.46		
**Parity**						
Primipara	477	93 (19.5)	1		1	
Multipara	915	238 (26.0)	1.45 (1.12–1.89)	0.005	1.38 (1.06–1.79)	0.017
**Mode of delivery**						
Spontaneous labour	619	159 (25.7)	1			
Primary (elective) C-section	189	36 (19.0)	0.70 (0.47–1.03	0.07		
Secondary (emergency) C-section	198	45 (22.7)	0.80 (0.55–1.16)	0.25		
Other/unknown	386	91 (23.6)	0.89 (0.66–1.19)	0.42		
**HIV RNA concentration (copies/ml)**						
<500	947	230 (24.3)	1			
>500	426	98 (23.0)	0.79 (0.56–1.10)	0.16		

* P-values were calculated using a logistic regression model including generalized estimating equations (GEE)

Table 3 abbreviations

OR: odds ratio; cART: combination antiretroviral therapy PI: Protease inhibitors; NNRTI: Non-nucleoside reverse-transcriptase inhibitors; NRTI: nucleoside reverse transcriptase inhibitors; BMI: body mass index; SSA: Sub-Saharan Africa; W. Europe: Western Europe; C-section: Caesarean section; Prim. C-section: primary Caesarean section (elective); Sec. C-section: secondary (emergency) Caesarean section

**Table 4:** Risk of a baby being born SGA <10th percentile. Multivariate analysis using a generalized estimation equation and stratified for PI- and NNRTI-based cART regimens.

	PI-based			NNRTI-based		
		Multivariate	P- Value*		Multivariate	P- Value*
	SGA (%)	OR (95% CI)		SGA (%)	OR (95% CI)	
**Total (n)**	928			438		
**Initiation of cART (n, %)**						
Postconception	138 (20.9)	1		42 (24.0)	1	
Preconception	77 (28.6)	1.49 (1.08, 2.10)	0.02	63 (24.0)	0.97 (0.62, 1.52)	0.9
**Region of origin**						
SSA	126 (23.2)	1		74 (25.1)	1	
Western Europe	36 (18.0)	0.73 (0.48–1.12)	0.2	18 (21.2)	0.85 (0.46–1.56)	0.6
Other	53 (28.6)	1.31 (0.89–1.92)	0.2	13 (22.4)	0.81 (0.40–1.68)	0.6
**Parity**						
Primipara	64 (19.3)	1		26 (19.0)	1	
Multipara	151 (25.3)	1.32 (0.96–1.82)	0.09	79 (26.2)	1.63	0.07

*P-values were calculated using a logistic regression model including generalized estimating equations (GEE)

Table 4 abbreviations

OR: odds ratio; cART: combination antiretroviral therapy PI: Protease inhibitors; NNRTI: Non-nucleoside reverse-transcriptase inhibitors; SSA: Sub-Saharan Africa

#### Neonatal birth weight

The median neonatal birth weight from HIV-positive mothers was 3.09 kg (IQR 2.702−3.405 kg), 0.33 kg lower than the median neonatal birth weight from (Generation R) HIV-negative mothers (3.42 kg [IQR 3.075−3.770 kg]) but just 0.030 kg lower Compared to neonates of women originating from sub-Saharan Africa this difference was 30 grams (lower) (S1 Table). The lowest median birth weight was found among the HIV-positive group of women who had received cART prior to conception (3.07 kg [IQR 2.637−3.384 kg]). When the type of cART regimen received by the HIV-positive women prior to conception was taken into consideration (Table 5), those receiving a PI-based regimen had the lowest median neonatal birth weight (3.02 kg [IQR 2.605−3.365 kg]). LBW (<2.5 kg) was found in 12.4% of all neonates from HIV-positive women. Among those HIV-positive women who began cART prior to conception, LBW was found in 14.2% of neonates compared with 11.3% of neonates in women who had started cART after conception (Table 5). Univariate analysis showed that the risk of LBW was found to be significantly greater among women who had begun cART prior to conception (1.35, 95% CI 1.01− 1.80, p = 0.05) compared to those beginning cART after conception. For the same comparison, but taking into account cigarette smoking, illicit drug use during pregnancy, parity and gestational age at birth, the risk of LBW was not statistically different (OR 1.34, (95% CI 0.94−1.92, p = 0.11) between women who started cART prior to conception vs those who began treatment after conception (S3 Table).

**Table 5:** Neonatal outcomes stratified for PI- and NNRTI-based regimens and based on whether cART was started before or after conception.

	Women receiving PI-based cART regimen				Women receiving NNRTI-based cART regimen			
	Total (n = 928) (%)	Pre- conception (n = 269) (%)	Post- conception (n = 659) (%)	P- value*	Total (n = 438) (%)	Pre- conception (n = 263) (%)	Post- conception (n = 175) (%)	P- value*
**SGA**								
No	713 (76.8)	192 (71.4)	521 (79.1)		333 (75.6)	200 (76.0)	133 (76.0)	
Yes	215 (23.2)	77 (28.6)	138 (20.9)	0.01	105 (24.4)	63 (24.0)	42 (24.0)	0.99
**Median birth weight (kg)**	3.08	3.02	3.1	0.03	3.14	3.14	3.11	0.91
IQR (kg)	2.715–3.399	2.605–3.365	2.770–3.425		2.698–3.44	2.69–3.405	2.7–3.5	
**Birth weight (n, %)**								
≥2.5 kg	791 (85.2)	222 (82.5)	569 (86.3)		362 (82.6)	213 (81.0)	149 (85.1)	
1.5−2.5 kg	114 (12.3)	40 (14.9)	74 (11.2)	0.12	55 (12.6)	34 (12.9)	21 (12.0)	0.68
<1.5 kg	23 (2.5)	7 (2.6)	16 (2.4)	0.80	21 (4.8)	16 (6.1)	5 (2.9)	0.11
**Median duration of pregnancy (weeks)**	39.00	39.00	39.00	0.61	39.14	39.14	39.57	0.05
IQR	38.0–40.1	37.9–40.0	38.0–40.1		37.8–40.3	37.7–40.3	38.0–40.4	
**Duration of pregnancy (n, %)**								
>37 weeks	801 (86.3)	224 (83.3)	577 (87.5)		365 (83.3)	216 (82.1)	149 (85.1)	
32−37 weeks	104 (11.2)	35 (13.0)	69 (10.5)	0.20	58 (13.2)	36 (13.7)	22 (12.6)	0.68
<32 weeks	23 (2.5)	10 (3.7)	13 (2.0)	0.10	15 (3.4)	11 (4.2)	4 (2.3)	0.27

* P-values were calculated using Mann-Whitney and Chi square tests.

Table 5 abbreviations

IQR: interquartile range; SGA: small for gestational age; cART: combination antiretroviral therapy PI: Protease inhibitors; NNRTI: Non-nucleoside reverse-transcriptase inhibitors

VLBW (<1.5 kg) occurred in 0.6% of neonates from (Generation R) HIV-negative women, and in 3.2% of infants from HIV-positive women (S1 Table). Among the HIV-positive women who started cART prior to conception, VLBW occurred in 4.4% of infants compared with 2.5% of infants from women who started cART after conception (p = 0.02) (Table 2). For the same comparison (cART initiation prior to and after conception) but corrected for smoking, illicit drug use during pregnancy, parity and gestational age at birth, the OR for VLBW was not significantly different between the two groups of women (OR 1.36, 95% CI 0.64−2.90 p = 0.42) (data not shown in the tables).

#### Preterm delivery

PTD (<37 weeks) occurred in 14.7% of infants from HIV-positive women, compared with only 5.2% among HIV-negative women (Generation R group); this percentage was marginally higher among HIV-negative women of SSA origin (5.5%) (S1 Table). PTD was significantly higher among HIV-positive women who began cART prior to conception (17.5%) compared with those who only began taking cART after conception (12.8%); as a result, the risk of a PTD was 1.38 times greater among women who took cART prior to conception vs those who took cART afterwards (95% CI 1.02−1.85, p = 0.04, univariate analysis). The same comparison, but taking into account type of cART regimen, maternal age, CD4^*+*^ cell count at delivery, trough CD4^+^ cell count, region of origin, smoking and illicit drug use showed that the risk of a PTD was 1.39 (95% CI 0.99−1.94, p = 0.06). Smoking and mode of delivery were significantly associated with PTD in multivariate analysis after stratification for cART drug regimen (S2 Table).

VPTD (<32 weeks) occurred in 0.6% of neonates from HIV-negative women compared with 2.8% from HIV-positive women. The rate of VPTD was significantly higher among women who had received cART prior to conception (4.0%) compared to those receiving cART after conception (2.0%, p = 0.02) (Table 2). The same comparison, corrected for type of drug regimen, maternal age at birth, region of origin, smoking, illicit drug use, CD4^+^ cell count and delivery mode, did not reveal any significant differences between the two groups (OR 1.25, 95% CI 0.86− 1.86, p = 0.22) (data not shown in the tables).

## Discussion

### Main findings

In our study of pregnant HIV-positive women receiving cART, the overall risk of SGA was 23.8%. By contrast, among the Dutch HIV-negative Generation R population, the overall risk of SGA was far lower (1.8%); similarly in the women of SSA origin (1.4%). The risk of SGA was significantly higher among women who had received cART prior to conception (27.3%) compared with those who had only begun taking cART after conception (21.5%). The risk of SGA was also higher among women (n = 928) receiving a PI-based cART (28.6%) prior to conception compared with those receiving the PI-based regimen after conception (20.9%, p = 0.01). In women receiving an NNRTI-based regimen (n = 438), however, there was no difference in the risk of SGA whether or not they received the regimen pre- or post-conception (24.0% vs 24.0%, p = 0.99).

### SGA and cART exposure

Few data exist describing a potential link between SGA and HIV infection, cART and PI-based regimens [7–11]. Of the studies which do exist, the data do not demonstrate any consistency [7,8,9,11]. One study found an increased risk of SGA in HIV-positive women who were not receiving cART [10].

Aaron *et al*.’s study, in the US, supports our findings. Among a cohort of 183 pregnant HIV-positive women, comprising 117 women receiving PIs and 39 receiving NNRTIs, there were high rates of SGA (<10th percentile, 31.2%) and (<3rd percentile, 12.6%). In Aaron *et al*.’s cohort, SGA was found to be associated with HIV disease severity, as well as with high rates of cigarette smoking (38%) and illicit drug use (35%). Women taking NNRTIs were less likely to have SGA infants below both the 10th and the 3rd percentile [7]. Most of the women receiving NNRTIs (74.9%) initiated cART during pregnancy, in contrast to our population. There was no significant association between SGA and cART initiated prior to pregnancy (25% of the cohort) or during pregnancy (p = 0.052). This could have been a power-problem due to small amount of women (46) women who started cART prior to conception. With a p value of p = 0.052, one could argue that there was a trend towards significance. Chen *et al*. found that SGA occurred at an overall rate of 18% among their population of 9504 pregnant HIV-positive women from Botswana and at a rate of 26.1% among those women who had begun cART prior to pregnancy and continued with it during pregnancy (n = 2851 on NNRTIs and n = 312 on PIs) [9]. Consistent with our findings, Chen *et al*. found a higher rate of SGA in HIV-positive women. They also found that women who initiated cART prior to pregnancy and continued with it during pregnancy had a higher risk of their infant being SGA (OR 1.3, 95% CI 1.1−1.5). In contrast to our findings, both CD4^+^ cell count and maternal hypertension were significantly associated with SGA. We found no association between SGA and CD4^+^ cell counts measured either at delivery or at nadir level. In total, 13.6% of CD4^+^ cell counts from all the women in Chen's cohort were below 200 cells/μl compared with just 4.3% of recorded CD4^+^ cell counts in our cohort. This could, in part, account for the lack of association in our cohort; with a median of 520 cells/μl our cohort appears to be immunologically stronger. Also consistent with our data, is a US cohort of 604 pregnancies among 477 HIV-positive women, with 222 women receiving PIs and 78 NNRTIs. This study also demonstrated a high rate of SGA infants below the 10th percentile (26%). Phiri *et al*. concluded that exposure to a PI during the first trimester of pregnancy actually lowered the risk of SGA (OR 0.54, 95% CI, 0.29−1.01) compared to non-PI-exposure throughout pregnancy [11]. This cohort was restricted to HIV-positive women who were eligible for Medicaid who were either multiparous or had disabilities and who represented the most disadvantaged population [11].

A French study (Briand *et al*.) investigated a cohort of 8192 singleton neonates, between 1990 and 2006, comprising 2630 women on PIs and 508 on NNRTIs. In contrast to our findings, no significant association was found between the type and duration of cART and the proportion of SGA infants (4%) [8]. However, Briand *et al*. did not compare cART that was initiated either before or during pregnancy.

### SGA and initiation of cART prior to conception

The cART regimens used in the above studies involved NNRTI-based and PI-based regimens, and most found an association between initiation of NNRTI prior to conception and risk of SGA [9]. The studies of Phiri and Briand did not define their populations by use of cART prior to conception [8, 11].

One hypothesis for reduced fetal growth, resulting in SGA, may be placental insufficiency due to vascular damage caused by HIV infection or cART-triggered endothelial injury [19]. Whether a specific cART regimen reduces endothelial injury associated with HIV infection or actually contributes to further endothelial cell activation is still unknown [20]. In our population, most women, whether they were receiving NNRTI- or PI-based regimens, had viral suppression, and only 4.3% of all women had a CD4+ cell count below 200 cells/μl. Our finding that cART initiated prior to pregnancy increases the risk of SGA suggests that cART may have a specific effect on fetal growth during the first trimester. One possible cause of adverse pregnancy outcomes on growth may be changes in the HIV-positive mother’s cytokine profile due to the influence of cART [9]. Chen *et al*. comment that successful pregnancy maintenance and HIV infection are both associated with Th type 2 cytokine predominance. As cART acts to reverse Th type 2 to Th type 1 cytokine predominance, this conversion could result in the modification of cytokine levels and immune milieu that may influence pregnancy outcome.

PIs are highly protein-bound and subject to backward transport through P-glycoprotein, so any adverse effects on fetal growth *in utero* cannot be explained by placental transfer, which is minimal or even non-existent [21]. A recent study on sex steroid hormones offered another hypothesis for exposure to PI and SGA [22]. Progesterone is essential for maintenance of pregnancy. Low levels have been associated with an increased incidence of pregnancy loss, placental abnormalities, prematurity and fetal growth restriction. These placental abnormalities could endanger fetal growth. Certain PIs, but not NNRTIs, cause reduced progesterone levels *in vitro* and in a pharmacological mouse model. Progesterone levels were also found to be lower in HIV-infected pregnant women receiving PI-based cART compared with uninfected controls. Lower progesterone levels correlated with fetal growth restriction in both the mouse model and in HIV-infected patients. *In vitro* trophoblast progesterone production was significantly reduced by lopinavir, atazanavir and ritonavir exposure, but not by darunavir [22].

We found that women who started on cART prior to the onset of their pregnancy had several differences in baseline characteristics (were older, a larger proportion had a CD4^+^ cell count below 200 cells/μl at time of cART initiation, were more often multiparous and more likely on an NNRTI regimen compared to women who started cART in pregnancy). Women on cART prior to pregnancy could have been in a more advanced disease stadium. Therefore, confounding could not be completely ruled out even though individual characteristics were corrected for in multivariate analyses. Neither median nadir CD4^+^ cell count nor median CD4^+^ cell count at the time of delivery were associated with an increased risk of SGA. This suggests that immunodeficiency is not the main cause of SGA as suggested in the paper by Aaron *et al*. [7].

#### Preterm delivery

Our study found a non-significant increased risk of PTD if cART was initiated prior to conception; PTD was also higher among women who smoked (multivariate analysis).

Previous studies on adverse birth outcomes related to cART use are diverse and the conflicting results may, in addition to the cART regimens used, be related to different populations at different disease stages, different settings (developed or resource-limited countries), limited access to cART regimens, small population numbers, lack of an HIV-negative control population and incomplete data [3, 4, 12, 14, 15, 23–28].

The strengths of this study were the detailed baseline demographic and clinical data recorded from a well-characterized large population in the ATHENA cohort. Our large sample of HIV-positive women allowed us to perform this research and to stratify for cART regimen used. Selection bias was not likely to occur as women from all social, ethnic and economic backgrounds have the same access to healthcare in the Netherlands. Women with other risk factors for SGA infants, such as illicit drug use, alcohol consumption or cigarette smoking, were in a minority. Our study has several limitations, mostly related to the retrospective nature of the study, resulting in missing data on smoking, substance abuse and socioeconomic background. This study covered several years of observation. Over time the guidelines for cART initiation changed and were based on different CD4-cell count levels. Fewer women were receiving an NNRTI-based regimen so the power to detect differences was reduced, so the finding of no significant association between SGA and preconception use of NNRTI may, in part, be due to sample size.

## Conclusion

In summary, our data showed that initiation of cART prior to conception is associated with an increased risk of SGA while cART initiated post-conception is not. When stratifying for cART regimen, this risk was clearly seen among women receiving PI-based regimens prior to conception who constituted the largest group. cART initiated prior to conception also revealed a trend towards significance in the increased risk of PTD.

As most of the current HIV guidelines advise initiating antiretroviral therapy at any CD4^+^ cell count, the number of pregnant cART users, who began their regimen prior to conception, will increase. Studies performed in this new population will be help to see if pre-conception cART and which regimen is the critical factor in the adverse pregnancy outcome. While the clinical benefits of cART regimens in HIV-infection and for PMTCT are clear, there remain risks for cART use prior to conception and during pregnancy [1, 29] which need further investigation. Further research involving larger cohorts of NNRTI users, who began their treatment prior to conception, is needed to clarify the potential association with SGA.

Fetal growth restriction, resulting in SGA, is a serious condition with increased morbidity and mortality, including neuro-developmental delay, as well as hypertension, obesity and diabetes mellitus in adulthood [30]. More information about the mechanisms underlying fetal growth restriction in HIV-infected pregnant women using cART is needed. Only when the potential impact of cART is fully understood can we determine the optimal individualised regimen for HIV-infected women of childbearing age.

## Supporting information

S1 Table Outcome of singleton infants born to HIV-negative, HIV negative of SSA descent, HIV-positive mothers. a = HIV-negative (Generation R) Solely to describe the non-HIV population in the Netherlands we selected 9778 children from HIV-negative control women who gave birth and were included in the Generation R study from 2000 to 2006. Exclusion criteria were a termination of pregnancy (n = 29), intra uterine fetal death (n = 75; 0.7%), HIV-positivity (n = 29), twin pregnancies (n = 262), missing data on birth weight (n = 77), an unknown gestational age (n = 2) or postnatal inclusion (n = 765). A total of 8539 singleton live births after at least 24 weeks gestation were included in the tables. Due to major differences in maternal characteristics between HIV- negative women and HIV positive women we chose to do our analysis in HEU only. The proportion of women of sub-Sahara African descend was higher in the HIV positive group 62.2% compared to the HIV negative group 9.6% [17].
(DOCX)

S2 Table Risk PTD (<37weeks gestation) univariate and multivariate analysis, GEE (generalized estimation equation). PTD: pre term delivery;cART: combination antiretroviral therapy; Origin: region of origin; SSA: Sub0Saharan Arfrica; SGA <10th: Small for gestational age <10th percentile; SGA <5th: Small for gestational age <5th percentile; IQR: interquartile range; C-section: Caesarean section; BMI: body mass index; PI: Protease inhibitors; NNRTI: Non-nucleoside reverse-transcriptase inhibitors; NRTI: nucleoside reverse transcriptase inhibitors.
(DOCX)

S3 Table Risk low birth weight (<2500 grams) univariate and multivariate analyis, GEE (generalized estimation equation). cART: combination antiretroviral therapy; Origin: region of origin; SSA: Sub0Saharan Arfrica; SGA <10th: Small for gestational age <10th percentile; SGA <5th: Small for gestational age <5th percentile; IQR: interquartile range; C-section: Caesarean section; BMI: body mass index; PI: Protease inhibitors; NNRTI: Non-nucleoside reverse-transcriptase inhibitors; NRTI: nucleoside reverse transcriptase inhibitors.
(DOCX)
