# Supplementary material for: Preconception use of cART by HIV-positive pregnant women increases the risk of infants being born small for gestational age
Source: PLoS One. 2018 Jan 19;13(1):e0191389. doi: 10.1371/journal.pone.0191389 (PMC5774764; doi:10.1371/journal.pone.0191389)
Supplement: S1 Table — a = HIV-negative (Generation R) Solely to describe the non-HIV population in the Netherlands we selected 9778 children from HIV-negative control women who gave birth and were included in the Generation R study from 2000 to 2006. Exclusion criteria were a termination of pregnancy (n = 29), intra uterine fetal death (n = 75; 0.7%), HIV-positivity (n = 29), twin pregnancies (n = 262), missing data on birth weight (n = 77), an unknown gestational age (n = 2) or postnatal inclusion (n = 765). A total of 8539 singleton live births after at least 24 weeks gestation were included in the tables. Due to major differences in maternal characteristics between HIV- negative women and HIV positive women we chose to do our analysis in HEU only. The proportion of women of sub-Sahara African descend was higher in the HIV positive group 62.2% compared to the HIV negative group 9.6% [17]. (DOCX) [file pone.0191389.s001.docx]

**Supplemental information 1**

**Table 1.** O**utcome of singleton infants born to HIV-negative, HIV negative of SSA descent, HIV-positive mothers.**

|  | **HIV-negative** | **HIV-negative SSA** | **HIV-positive** |
| --- | --- | --- | --- |
|  | **Generation R^a^** | **Generation R -** | **SHM** |
|  | **n=8539 (%)** | **n=900 (%)** | **n=1392 (%)** |
| **Median birth weight (kg)** |  |  |  |
| kilogram | 3.420 | 3.120 | 3.090 |
| IQR | 3.070-3.765 | 2.900-3.498 | 2.702-3.405 |
| **SGA <10th (n, %)** |  |  |  |
| No | 8387 (98.2) | 887 (98.6) | 1061 (76.2) |
| Yes | 152 (1.8) | 13 (1.4) | 331 (23.8) |
| **SGA <5th (n, %)** |  |  |  |
| No |  |  | 1199 (86.1) |
| Yes |  |  | 193 (13.9) |
| **Birth weight (n, %)** |  |  |  |
| ≥2.5 kg | 8124 (95.1) | 886 (98.4) | 1174 (84.3) |
| 1.5−2.5 kg | 368 (4.3) | 9 (1.0) | 173 (12.4) |
| <1.5 kg | 47 (0.6) | 5 (0.6) | 45 (3.2) |
| **Duration of** |  |  |  |
| **pregnancy (weeks)** | 40.00 | 39.71 | 39.14 |
| IQR | 39.14-40.86 | 38.71-40.71 | 38.00-40.14 |
| **Pregancy duration** |  |  |  |
| >37 weeks | 8098 (94.8) | 850 (94.4) | 1188 (85.3) |
| <37 weeks | 391 (4.6) | 46 (5.1) | 165 (11.9) |
| <32 weeks | 50 (0.6) | 4 (0.4) | 3 (2.8) |
| **Perinatal death** |  |  |  |
| No/unknown |  |  | 1374 (98.7) |
| Yes |  |  | 18 (1.3) |

Table 1 legend

^a^= HIV-negative (Generation R) Solely to describe the non-HIV population in the Netherlands we selected 9778 children from HIV-negative control women who gave birth and were included in the Generation R study from 2000 to 2006. Exclusion criteria were a termination of pregnancy (n=29), intra uterine fetal death (n=75; 0.7%), HIV-positivity (n=29), twin pregnancies (n=262), missing data on birth weight (n=77), an unknown gestational age (n=2) or postnatal inclusion (n=765). A total of 8539 singleton live births after at least 24 weeks gestation were included in the tables. Due to major differences in maternal characteristics between HIV- negative women and HIV positive women we chose to do our analysis in HEU only. The proportion of women of sub-Sahara African descend was higher in the HIV positive group .62.2 % compared to the HIV negative group 9.6 % [17].
