# Supplementary material for: Preconception use of cART by HIV-positive pregnant women increases the risk of infants being born small for gestational age
Source: PLoS One. 2018 Jan 19;13(1):e0191389. doi: 10.1371/journal.pone.0191389 (PMC5774764; doi:10.1371/journal.pone.0191389)
Supplement: S2 Table — PTD: pre term delivery;cART: combination antiretroviral therapy; Origin: region of origin; SSA: Sub0Saharan Arfrica; SGA <10th: Small for gestational age <10th percentile; SGA <5th: Small for gestational age <5th percentile; IQR: interquartile range; C-section: Caesarean section; BMI: body mass index; PI: Protease inhibitors; NNRTI: Non-nucleoside reverse-transcriptase inhibitors; NRTI: nucleoside reverse transcriptase inhibitors. (DOCX) [file pone.0191389.s002.docx]

**Supplemental information 2**

**Table 2. Risk PTD (<37weeks gestation) univariate and multivariate analysis, GEE
(generalized estimation equation).**

| **PTD** | **n** | **PTD** | **Univariate** | **P-** | | **Multivariate** | **P-** |  |
| --- | --- | --- | --- | --- | --- | --- | --- | --- |
|  |  | **n (%)** | **OR (95% CI)** | **value** | | **OR (95% CI)** | **value** |  |
| **Initiation cART** |  |  |  |  | |  |  |  |
| postconception | 842 | 107 (12.7) | 1 |  | |  |  |  |
| preconception | 550 | 96 (17.5) | 1.38 (1.02-1.85) | 0.04 | | 1.39 (0.99-1.94) | 0.06 |  |
| **cART regimen** |  |  |  |  | |  |  |  |
| PI | 928 | 126 (13.6) | 1 |  | |  |  |  |
| NNRTI | 438 | 73 (16.7) | 1.30 (0.95-1.77) | 0.11 | |  |  |  |
| Both / NRTI only | 26 | 4 (16.0) | 1.15 (0.41-3.19) | 0.78 | |  |  |  |
| **Age at delivery** |  |  |  |  | |  |  |  |
|  |  |  | 1.02 (1.00-1.05) | 0.10 | | 1.01 (0.98-1.04) | 0.35 |  |
| **BMI** |  |  |  |  | |  |  |  |
|  |  |  |  |  | |  |  |  |
|  |  |  | 0.97 (0.92-1.03) | 0.33 | |  |  |  |
| **Maternal CD4***^+^* **concentration at delivery (cells/µl)** |  |  |  |  | |  |  |  |
| ≥500 | 738 | 106 (14.4) | 1 |  | | 1 |  |  |
| 200-500 | 570 | 82 (14.4) | 0.89 (0.72-1.34) | 0.92 | | 1.01 (0.73-1.40) | 0.96 |  |
| <200 | 60 | 14 (23.3) | 1.77 (0.55-3.32) | 0.08 | | 1.78 (0.90-3.51) | 0.10 |  |
| **Nadir CD4^+^** |  |  |  |  | |  |  |  |
| ≥500 | 258 | 34 (13.2) | 1 |  | |  |  |  |
| 200-500 | 643 | 81 (12.6) | 0.95 (0.59-1.51) | 0.83 | |  |  |  |
| <200 | 491 | 88 (17.9) | 1.38 (0.86-2.28) | 0.18 | |  |  |  |
| **HIV RNA concentration (copies/ml)** |  |  |  |  | |  |  |  |
| ≤500 | 947 | 137 (14.5) | 1 |  | |  |  |  |
| >500 | 426 | 64 (15.0) | 1.01 (0.68-1.52) | 0.95 | |  |  |  |
| **Region of origin** |  |  |  |  | |  |  |  |
| SSA | 853 | 128 (15.0) | 1 |  | |  |  |  |
| W. Europe | 288 | 33 (11.5) | 0.71 (0.45-1.11) | 0.14 | |  |  |  |
| Other | 251 | 42 (16.7) | 1.13 (0.75-1.70) | 0.51 | |  |  |  |
| **Smoking** |  |  |  |  | |  |  |  |
| No | 643 | 77 (12.0) | 1 |  | | 1 |  |  |
| Yes | 114 | 27 (23.7) | 2.31 (1.41-3.79) | 0.001 | | 2.87 (1.60-5.16) | 0.0004 |  |
| **unknown** | 635 | 99 (15.6) | 1.42 (1.04-1.95) | 0.03 | | 1.01 (0.49-2.06) | 0.98 |  |
| **Alcohol** |  |  |  |  | |  |  |  |
| No | 664 | 90 (13.6) | 1 |  | |  |  |  |
| Yes | 75 | 8 (10.7) | 0.77 (0.36-1.66) | 0.50 | |  |  |  |
| unknown | 653 | 105 (16.1) | 1.26 (0.94-1.71) | 0.12 | |  |  |  |
| **Drugs** |  |  |  |  | |  |  |  |
| No | 718 | 91 (12.9) | 1 |  | |  |  |  |
| Yes | 21 | 6 (27.3) | 2.28 (0.91-5.71) | 0.08 | | 1.58 (0.52-4.74) | 0.41 |  |
| unknown | 299 | 106 (16.0) | 1.30 (0.97-1.75) | | 0.08 | 1.31 (0.67-2.57) | 0.43 | |
| **Parity** |  |  |  | |  |  |  | |
| Primi | 477 | 76 (15.9) | 1 | |  |  |  |  |
| Multi | 915 | 127 (13.9) | 0.87 (0.64-1.17) | | 0.37 |  |  |  |
| **Gender** |  |  |  | |  |  |  | |
| Male | 717 | 114 (15.9) | 1 | |  |  |  |  |
| Female | 674 | 89 (13.2) | 0.83 (0.62-1.10) | | 0.17 |  |  |  |
| **Mode of delivery** |  |  |  | |  |  |  | |
| Spontaneous labour | 619 | 47 (7.6) | 1 | |  | 1 |  | |
| Primary (elective) C-section | 189 | 46 (24.3) | 3.72 (2.37-5.83) | | <0.0001 | 4.06 (2.56-6.43) | <0.0001 | |
| Secondary (emergency) C-section | 198 | 47 (23.7) | 3.83 (2.50-5.86) | | <0.0001 | 4.09 (2.64-6.33) | <0.0001 | |
| Unknown | 386 | 63 (16.3) | 2.44 (1.69-3.51) | | <0.0001 | 2.21 (1.36-3.60) | 0.001 | |

Legend supplemental table 2

PTD: pre term delivery;cART: combination antiretroviral therapy; Origin: region of origin; SSA: Sub0Saharan Arfrica; SGA <10th: Small for gestational age <10th percentile; SGA <5th: Small for gestational age <5th percentile; IQR: interquartile range; C-section: Caesarean section; BMI: body mass index; PI: Protease inhibitors; NNRTI: Non-nucleoside reverse-transcriptase inhibitors; NRTI: nucleoside reverse transcriptase inhibitors.
