# Supplementary material for: Preconception use of cART by HIV-positive pregnant women increases the risk of infants being born small for gestational age
Source: PLoS One. 2018 Jan 19;13(1):e0191389. doi: 10.1371/journal.pone.0191389 (PMC5774764; doi:10.1371/journal.pone.0191389)
Supplement: S3 Table — cART: combination antiretroviral therapy; Origin: region of origin; SSA: Sub0Saharan Arfrica; SGA <10th: Small for gestational age <10th percentile; SGA <5th: Small for gestational age <5th percentile; IQR: interquartile range; C-section: Caesarean section; BMI: body mass index; PI: Protease inhibitors; NNRTI: Non-nucleoside reverse-transcriptase inhibitors; NRTI: nucleoside reverse transcriptase inhibitors. (DOCX) [file pone.0191389.s003.docx]

**Supplemental information 3**

**Table 3. Risk low birth weight (<2500 grams) univariate and multivariate analyis, GEE (generalized estimation equation).**

| **LBW** | **n** | **LBW** | **Univariate** | **P-** | **Multivariate** | **P-** |
| --- | --- | --- | --- | --- | --- | --- |
|  |  | **n (%)** | **OR (95% CI)** | **value** | **OR (95% CI)** | **value** |
| **Initiation cART** |  |  |  |  |  |  |
| Postconception | 842 | 116 (13.8) | 1 |  | 1 |  |
| Preconception | 550 | 102 (18.5) | 1.35 (1.01-1.80) | 0.05 | 1.34 (0.94-1.92) | 0.11 |
| **Type of cART regimen** |  |  |  |  |  |  |
| PI-based | 928 | 137 (14.8) | 1 |  |  |  |
| NNRTI-based | 438 | 76 (17.4) | 1.19 (0.88-3.97) | 0.26 |  |  |
| Both/NRTI only | 26 | 5 (20.0) | 1.47 (0.54-3.97) | 0.45 |  |  |
| **Age at delivery** |  |  |  |  |  |  |
|  |  |  | 1.01 (0.99-1.05) | 0.37 |  |  |
| **BMI** |  |  |  |  |  |  |
|  |  |  | 1.03 (0.92-1.03) | 0.41 |  |  |
| **Maternal CD4***^+^* **concentration at delivery (cells/µl)** |  |  |  |  |  |  |
| ≥500 | 738 | 113 (15.0) | 1 |  |  |  |
| 200−500 | 570 | 87 (15.3) | 0.99 (0.75-1.35) | 0.99 |  |  |
| <200 | 60 | 15 (25.0) | 1.60 (0.81-3.19) | 0.17 |  |  |
| **Nadir maternal CD4^+^ concentration (cells/µl)** |  |  |  |  |  |  |
| ≥500 | 258 | 32 (12.4) | 1 |  |  |  |
| 200-500 | 643 | 104 (16.2) | 1.40 (0.89-2.27) | 0.14 |  |  |
| <200 cells | 491 | 82 (16.7) | 1.43 (0.89-2.32) | 0.14 |  |  |
| **HIV RNA concentration (copies/ml)** |  |  |  |  |  |  |
| <500 | 947 | 157 (16.6) | 1 |  |  |  |
| >500 | 426 | 60 (14.1) | 0.83 (0.55-1.23) | 0.33 |  |  |
| **Region of origin** |  |  |  |  |  |  |
| SSA | 853 | 134 (15.7) | 1 |  |  |  |
| W. Europe | 288 | 34 (11.8) | 0.73 (0.48-1.13) | 0.15 |  |  |
| Other | 251 | 50 (19.9) | 1.32 (0.91-1.95) | 0.14 |  |  |
| **Smoking** |  |  |  |  |  |  |
| No | 643 | 95 (14.8) | 1 |  | 1 |  |
| Yes | 114 | 26 (22.8) | 1.68 (1.03-2.74) | 0.04 | 0.93 (0.50-1.71) | 0.82 |
| unknown | 635 | 97 (15.3) | 1.06 (0.78-1.43) | 0.71 | 0.85 (0.38-1.89) | 0.69 |
| **Alcohol** |  |  |  |  |  |  |
| No | 664 | 104 (15.7) | 1 |  |  |  |
| Yes | 75 | 11 (14.7) | 1.03 (0.77-1.38) | 0.85 |  |  |
| unknown | 653 | 103 (15.8) | 0.91 (0.47-1.78) | 0.80 |  |  |
| **Drugs** |  |  |  |  |  |  |
| No | 718 | 107 (15.2) | 1 |  | 1 |  |
| Yes | 22 | 7 (31.8) | 2.22 (0.86-5.75) | 0.10 | 1.71 (0.63-4.65) | 0.29 |
| unknown | 299 | 104 (15.7) | 1.05 (0.80-1.40) | 0.69 | 1.03 (0.47-2.27) | 0.94 |
| **Parity** |  |  |  |  |  |  |
| Primipara | 477 | 83 (17.4) | 1 |  | 1 |  |
| Multipara | 915 | 135 (14.8) | 0.77 (0.58-1.02) | 0.06 | 0.67 (0.47-0.97) | 0.03 |
| **Duration of pregnancy (weeks)** |  |  |  |  |  |  |
| >37 | 1045 | 40 (3.8) | 1 |  | 1 |  |
| <37 | 334 | 167 (50) | 24 (16.3-35.0) | <0.0001 | 24.48 | <0.0001 |
|  |  |  |  |  | (16.49-36.33) |  |
| <32 | 13 | 11 (84.6) | 120 (29.5- 486) | <0.0001 | 108.09 | <0.0001 |
|  |  |  |  |  | (25.34-461.23) |  |
| **Gender** |  |  |  |  |  |  |
| Male | 717 | 116 (16.2) | 1 |  |  |  |
| Female | 674 | 102 (15.1) | 0.96 (0.73-1.27) | 0.59 |  |  |
| **Mode of delivery** |  |  |  |  |  |  |
| Spontaneous labour | 619 | 66 (10.7) | 1 |  | 1 |  |
| Primary (elective) C-section | 189 | 44 (23.3) | 2.48 (1.63-3.78) | <0.001 | 3.28 (1.41-7.77) | 0.006 |
| Secondary (emergency) C-section | 198 | 47 (23.7) | 2.58 (1.72-3.86) | <0.001 | 4.52 (2.22-9.11) | <0.0001 |
| Other/unknown | 386 | 61 (15.8) | 1.56 (1.12-2.22) | 0.009 | 2.24 (0.88-5.75) | 0.09 |

Legend supplemental table 3

cART: combination antiretroviral therapy; Origin: region of origin; SSA: Sub0Saharan Arfrica; SGA <10th: Small for gestational age <10th percentile; SGA <5th: Small for gestational age <5th percentile; IQR: interquartile range; C-section: Caesarean section; BMI: body mass index; PI: Protease inhibitors; NNRTI: Non-nucleoside reverse-transcriptase inhibitors; NRTI: nucleoside reverse transcriptase inhibitors.
